# Identifying radiation-induced symptoms from an interview survey

**DOI:** 10.1093/jrr/rraf014

**Published:** 2025-03-31

**Authors:** Kenichi Yokota, Mariko Mine, Noboru Takamura, Yasushi Miyazaki

**Affiliations:** Biostatistics Section, Division of Scientific Data Registry, Atomic Bomb Disease Institute, 1-12-4 Sakamoto, Nagasaki University 852-8523, Nagasaki, Japan; Biostatistics Section, Division of Scientific Data Registry, Atomic Bomb Disease Institute, 1-12-4 Sakamoto, Nagasaki University 852-8523, Nagasaki, Japan; Biostatistics Section, Division of Scientific Data Registry, Atomic Bomb Disease Institute, 1-12-4 Sakamoto, Nagasaki University 852-8523, Nagasaki, Japan; Department of Hematology, Atomic Bomb Disease Institute, 1-12-4 Sakamoto, Nagasaki University 852-8523, Nagasaki, Japan

**Keywords:** atomic bomb survivors, atomic bomb disaster survey, acute radiation syndrome, external injuries, burns

## Abstract

Studies on the atomic bomb have reported a relatively high incidence of acute symptoms, even at below the threshold dose of radiation, and are therefore assumed to include symptoms caused by non-radiation factors. In this study, to investigate the influence of external injuries and burns on symptom expression and the possibility of distinguishing radiation-induced symptoms, we reanalysed data from the survey conducted immediately after the atomic bombing of Nagasaki. The adjusted odds ratios (ORs) of radiation per 1 Gy for the occurrence of 16 symptoms ranged from 1.14 to 1.46, based on sex, age at the time of the bombing, radiation dose, external injuries, and burns. This study also included 243 deaths, and thus provides information not seen in other studies, such as the frequency of symptoms in deaths and ORs for symptom occurrence. However, the adjusted ORs for external injuries or burns were smaller than the unadjusted ORs, suggesting that external injuries and burns confound the development of radiation-induced symptoms. Symptom data obtained from interviews such as those used in this study may not be appropriate for use alone because such data include non-radiation factors. Radiation-induced symptoms are often considered to be a syndrome, and the multiple correspondence analyses also revealed that high-dose exposure is associated with nausea and vomiting, subsequent epilation and bleeding tendency as a bone marrow disorder, and inflammation symptoms due to a weakened immune system. Thus, radiation exposure may be indicated by not just one, but rather, a combination of symptoms.

## INTRODUCTION

The Atomic Bomb Disaster Survey was conducted in Nagasaki immediately after the atomic bombing on 9 August 1945. The original survey document is currently stored at Nagasaki University. The survey was proposed by Colonel E. DeCoursey of the United States Army Investigation Team and conducted by its director, Professor Raisuke Shirabe of the former Nagasaki Medical College, under the direction of Professor Masao Tsuzuki of the Imperial University of Tokyo [1]. The survey was conducted on ~6000 people with the help of ~50 medical doctors and students over a period of just over a month. The survey report prepared by Prof. Shirabe summarizes descriptive statistics on four main categories: mortality, survival, surgical injuries, and radiation sickness. The frequency, timing, and relationship to external injury of 11 symptoms by sex, age, distance, shielding, and external injuries were tabulated and discussed in relation to radiation. Furthermore, the report considered the possibility that factors other than radiation, such as contaminated drinking water or food, might also cause similar symptoms [2]. The symptoms associated with atomic bomb exposure are the result of a complex mixture of factors, not just solely radiation. An English version of this report, which has been summarized and revised by American physicians, was published in 1953 [3]. Repeated analyses have also been conducted as part of the Life Span Study [4–8], a research project of the Radiation Effects Research Foundation (RERF). Of these analyses, a study utilizing data on acute radiation symptoms has also been conducted. However, few studies have evaluated symptoms [9–11], and most have been conducted with the purpose of correcting estimated radiation doses [12–14]. Data on acute symptoms were based on interviews and may not have exclusively captured symptoms induced by radiation. Consequently, the association between radiation exposure and symptom onset has not been sufficiently evaluated. Acute radiation symptoms are considered to be tissue reactions that do not occur below a threshold dose [15]. In general, the majority of acute radiation symptoms are observed at doses of ≥1 Sv (Gy) [16, 17]. By contrast, data on symptoms observed in the survey conducted after the atomic bombing indicated that the frequency of onset below the threshold dose may not be negligible, thereby suggesting that non-radiation factors may be involved. In addition to radiation, other factors, such as sanitary conditions and infectious diseases, should also be considered. As with radiation, blast and heat rays are more intense at close range, and thus, external injuries and burns may be confounding factors in the development of symptoms. To the best of our knowledge, no attempts have been made to conduct a mutually coordinated analysis of the development of external injuries, burns, and radiation symptoms in the human body as a result of the blast, heat rays, and radiation generated by atomic bomb explosions. Therefore, in the present study, we conducted an analysis in which we adjusted for these factors.

## MATERIALS AND METHODS

This study was reviewed and approved by the Institutional Ethics Committee of Nagasaki University Graduate School of Biomedical Sciences (No. 22102801). The original Atomic Bomb Disaster Survey forms used in this study were donated to RERF by Mrs. Junko Shirabe, the wife of Prof. Shirabe, in October 1999, and transferred from RERF to Nagasaki University in August 2015. We performed an analysis using anonymized data and announced the aims and procedures to the public (https://mdp.nagasaki-u.ac.jp/images/research/disclosure3/3-05.pdf). All methods were performed in accordance with relevant guidelines and regulations.

### Preparation of atomic bomb disaster survey data for analysis

The Atomic Bomb Disaster Survey was conducted from 29 October to 20 November 1945, immediately after the atomic bombing, and covered ~6000 victims within ~4 km of the hypocenter, with care taken to avoid regional bias. The survey was based on a questionnaire prepared in advance and consisted of door-to-door visits and group interviews at factories and schools. The questionnaire included items on personal attributes such as name, date of birth, age at the time of the survey, and address (no question items on gender), as well as detailed information on the location of the victim (address and name of facility) at the time of the bombing, distance from the hypocenter, map coordinates, and exposure-related conditions, such as outdoor, indoor, or bomb shelter, shelter material, type, amount and color of clothing, and body direction and posture in relation to the hypocenter. The survey also included detailed information on human injuries, including external injuries (type, location, and extent of injuries, such as glass wounds, bruises, and fractures), burns (location, size, and severity), and acute symptoms, such as (i) consciousness disturbance, (ii) dizziness, (iii) headache, (iv) nausea, (v) vomiting, (vi) abdominal pain, (vii) diarrhea, (viii) fever, (ix) herpes, (x) cramps, (xi) other radiation symptoms, (xii) appetite loss, (xiii) malaise, (xiv) subcutaneous, gingivitis, nasal, and conjunctival bleeding tendencies, hemoptysis, hematemesis, melena, and hematuria (sub-classifications of this category), (xv) tonsillitis or dysphagia, (xvi) epilation with the sites of hair, eyebrows, beard, armpits, pubic hair and degree of loss, (xvii) skin pigmentation, (xviii) anhidrosis, (xix) menstrual irregularities, (xx) pregnancy, (xxi) miscarriage, (xxii) libido, and (xxiii) other symptoms, as well as medical treatment and health progress.

**Fig. 1:**
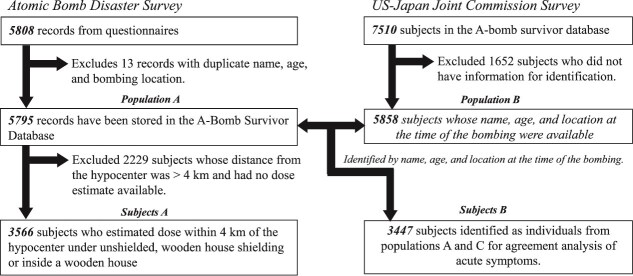
**Flowchart of subject selection**. **Population A, which comprised all of the respondents in the Atomic Bomb Disaster Survey, was used to show the geographic distribution of the subjects in**  **Fig. 1**  **and**  **Table 1****. Population B was selected for an agreement analysis of symptoms from the respondents of the Joint Commission Survey. Subjects A were the main subjects in this study and were used for the analysis based on radiation dose in**  **Table 4****. Data exclusion from population A is shown in**  **Table 4****. Subjects B were identified as the same individuals between populations A and B, but two subjects who did not have information about their symptoms were excluded.**

Basic information such as sex and age, distance from the hypocenter as a parameter for dose estimation, and shielding conditions were reclassified according to the relevant items in the questionnaire. Although the questionnaire did not include any items on sex, we considered this essential as a basic characteristic. Therefore, based on information from the database of ~150 000 atomic bomb survivors, we created a sex table of first names that were more common among males and females at the time, and applied it to names that were largely divided between the sexes. The occupation and clothing columns were also used as a reference for sex. Monpe, loose agricultural work trousers worn mainly by women, and the national jacket, worn mainly by men, were used to identify sex, and those for whom insufficient information could be obtained were considered unknown. The questionnaire included items for the date of birth and age at the time of the survey. The age at the time of the bombing (ATB) was calculated from the date of birth. For those who did not provide a date of birth (~25% of the total), age at the time of the survey was used as age ATB. Distance from the hypocenter was calculated from map coordinates of the investigator based on the location of the respondents. Three types of shielding for which doses can be calculated using the ABS93D dosimetry system are: unshielded, in the shade of wooden houses or trees, and inside wooden houses. Inside buildings with slate roofs that did not fall into these categories were reclassified as ‘other shielding’. Those with descriptions in the ‘hypocenter shielding’ column under ‘unshielded’ were reclassified as ‘other shielding’. From the total questionnaire data for 5808 participants, data were generated for 5795 after excluding 13 cases considered duplicates based on name, date of birth (age), and location ATB (Fig. 1). Thus, the data contain 58 fewer persons than the preliminary report [2]. Reclassification reduced the number of survivors, females, those aged <20 years, those <1.5 km from the hypocenter, unshielded, and exposed in wooden houses (Table 1).

**Table 1:** Characteristics of the survey subjects and difference in numbers between the present study and the previous report by Professor Shirabe

Characteristic		Present study	(%)	Shirabe report^a^	(%)	Differences
Status	Alive	5462	(94.3)	5520	(94.3)	−58
	Deceased	333	(5.7)	333	(5.7)	0
Sex	Male	2619	(45.2)	2617	(44.7)	2
	Female	3161	(54.5)	3236	(55.3)	−75
	Unknown	15	(0.3)	0		15
Age at the time of the bombing (years)	< 10	483	(8.3)	558	(9.5)	−75
	10–19	2969	(51.2)	3048	(52.1)	−79
	20–29	709	(12.2)	646	(11.0)	63
	30–39	476	(8.2)	484	(8.3)	−8
	40–49	526	(9.1)	527	(9.0)	−1
	50–59	393	(6.8)	369	(6.3)	24
	60–86	239	(4.1)	221	(3.8)	18
Distance from the hypocenter (km)	< 1.0	476	(8.2)	635	(10.8)	−159
	1.0–1.4	1273	(22.0)	1506	(25.7)	−233
	1.5–1.9	1126	(19.4)	884	(15.1)	242
	2.0–2.9	1741	(30.0)	1749	(29.9)	−8
	3.0–3.9	1103	(19.0)	1079	(18.4)	24
	4.0–5.9	76	(1.3)	0		76
Shielding	Outdoors, open^b^	430	(7.4)	613	(10.5)	−183
	Outdoors, wooden house shielding^b^	119	(2.1)	0		119
	Outdoors, others shielding	680	(11.7)	707	(12.1)	−27
	Indoors, wooden house^b^	3039	(52.4)	3382	(57.8)	−343
	Indoors, concrete/brick building	812	(14.0)	820	(14.0)	−8
	Bomb shelters/dugouts	348	(6.0)	331	(5.7)	17
	Indoors, other materials	190	(3.3)	0		190
	Unknown	177	(3.1)	0		177
Total		5795	(100.0)	5853	(100.0)	−58

^a^See reference [2].

^b^Shielding types for which a radiation dose estimation was available.

These valuable survey data have been registered in the Atomic Bomb Survivors Database [18] for future preservation and use as an archive.

### Reliability assessment for symptom data

To confirm the reliability of the response data, we evaluated whether each subject gave consistent responses in the Atomic Bomb Disaster Survey and the US–Japan Joint Commission Survey conducted at the same time. The US–Japan Joint Commission Survey was conducted in Hiroshima and Nagasaki by a survey team headed by Colonel A. W. Oughterson of the United States Army, with Professor Masao Tsuzuki of the University of Tokyo serving as the Japanese representative. The survey of symptoms appears to have been conducted between September and November 1945, based on the dates on the survey questionnaire. It is noted that the Nagasaki Joint Commission Survey was conducted in cooperation with Professor Sawada of Kyushu Imperial University and Prof. Shirabe of Nagasaki Medical College [19]. Although the relationship between the two surveys is unclear, many of the items in these surveys are similar. The Nagasaki Joint Commission Survey included 7510 persons. In 1998, the Atomic Bomb Disease Institute of Nagasaki University registered the acute symptoms of the subjects in the Nagasaki Joint Commission Survey in the A-Bomb Survivor Database. The 5858 cases with name, age, and location ATB in the Nagasaki Joint Commission Survey were matched with those in the Atomic Bomb Disaster Survey to identify 3449 (58.9%) persons. Symptom occurrence was compared for the 3447 subjects, excluding two subjects with missing symptom data, to evaluate the reliability of the symptom data (Fig. 1).

### Estimation of exposed radiation dose

The Nagasaki atomic bomb version of the ABS93D system originally developed at Hiroshima University [20] has been used to estimate individual radiation doses according to distance from the hypocenter and type of shielding. Dose estimates are available for a distance of ≤4 km from the hypocenter, and for three types of shielding, as mentioned in the previous section. The Atomic Bomb Disaster Survey also included a considerable number of individuals who were situated within concrete and brick buildings and in bomb shelters. However, it is not possible to estimate the radiation doses for individuals who were protected by these types of shielding. Of the 5795 individuals, we were able to estimate radiation doses for 3566 after excluding those whose sex was unknown and those whose distance was >4 km. Table 2 shows the number of persons in each dose category by life and death. In total, 83.1% of the deceased were categorized as being exposed to doses of ≥0.5 Gy, and 79.4% of the survivors were categorized as being exposed to doses <0.5 Gy.

**Table 2:** Characteristic of subjects for the analysis related to radiation dose by life-or-death status at the time of the survey

Radiation dose	Alive	(%)	Deceased	(%)	Total
< 0.005 Gy	1015^a^	(99.6)	4	(0.4)	1019
0.005–	1048^a^	(99.0)	11	(1.0)	1059
0.1–	128^a^	(98.5)	2	(1.5)	130
0.2–	449^a^	(94.9)	24	(5.1)	473
0.5–	210	(95.0)	11^b^	(5.0)	221
1.0–	262	(88.8)	33^b^	(11.2)	295
2.0–	113	(81.9)	25^b^	(18.1)	138
5.0–	98	(42.4)	133^b^	(57.6)	231
Total	3323	(93.2)	243	(6.8)	3566

^a^The total number of survivors exposed to a radiation dose <500 mGy was 2640(79.4%).

^b^The total number of deceased exposed to a radiation dose ≥500 mGy was 202 (83.1%).

### Analysis of external injuries, burns, and radiation symptoms

The frequency of external injuries, burns, and radiation symptoms by dose category was examined for the 3566 subjects analysed for whom radiation doses could be estimated. The numbers of subjects who reported subcutaneous bleeding, petechiae, gingivitis, and nosebleeds were relatively low, so these symptoms were classified as bleeding tendency. In addition, stomatitis, gingivitis, sore throat, tonsillitis, and dysphagia were classified as inflammation symptoms. The analysis was performed on 16 symptoms: consciousness disturbance, dizziness, headache, nausea, vomiting, abdominal pain, diarrhea, fever, herpes, cramps, bleeding tendency, inflammation symptoms, epilation, skin pigmentation, appetite loss, and malaise. Each of these symptoms was categorized according to the frequency of occurrence by radiation dose. The mechanism of onset of epilation is well known. To determine whether epilation was caused by radiation, the frequency of onset and the duration of epilation were tabulated.

### Combination of symptoms

A previous report stated that diarrhea and fever often appeared concurrently [2]. In the present study, we also analysed the radiation dose and combinations of 16 symptoms, which have not been studied in detail. The relationship between radiation dose and each symptom was visualized, with adjustments for other factors.

### Statistical analysis

Kappa coefficients [21] as a measure of reliability were used to compare the presence or absence of each symptom for subjects in both the Atomic Bomb Disaster Study and the US–Japan Joint Commission Survey. A logistic regression model was used for multivariate analysis of the effect of radiation dose on symptom onset. For each symptom, we used symptom onset as the outcome, adjusted for sex (male and female) and age ATB (years), and obtained ORs for three factors: radiation dose (Gy), presence or absence of external injuries, and burns. Multiple correspondence analysis for spatial arrangement was used to analyse symptom combinations with the presence or absence of 16 symptoms, external injuries, burns, life or death, and categorized radiation dose (eight categories: < 5 mGy, 5–100 mGy, 100–200 mGy, 200–500 mGy, 0.5–1 Gy, 1–2 Gy, 2–5 Gy, and ≥ 5 Gy). SAS 9.4 [22] was used for all analyses.

## RESULTS

### Geographical distribution of the survey subjects

**Fig. 2:**
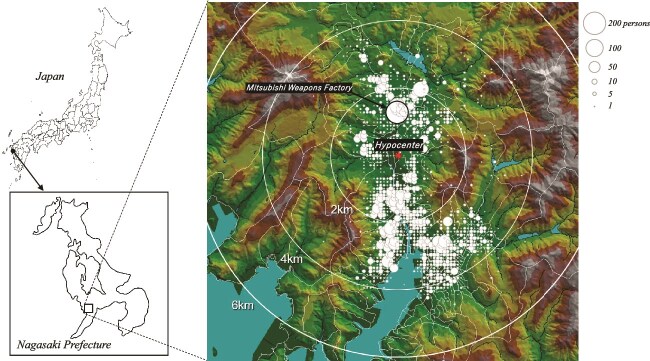
Geographical distribution of survey subjects at the time of the bombing.

Figure 2 shows the geographic distribution of the locations of the 5795 survey participants ATB. The size of the circles indicates the number of persons. The largest circle north of the hypocenter represents the number of survivors at the Mitsubishi arms factory. The survey subjects were located in residential areas between 1 and 4 km from the hypocenter, excluding areas near the hypocenter where survivors were rare, but people were concentrated in factories. In particular, a concentration of ~200 people was seen at the Mitsubishi arms factory ~1.5 km north of the hypocenter. The age distribution of the participants shows that those aged 10–19 years accounted for about half of all subjects (Table 1), suggesting that many minor schoolchildren were mobilized to work in factories at that time.

### Reliability of symptom data

Table 3 shows the results of a comparison of responses to two surveys conducted at the same time, the Atomic Bomb Disaster Survey and the US–Japan Joint Commission Survey. As can be seen, 13 symptoms were compared. In addition, there were similar items for four other symptoms (appetite loss, malaise, anhidrosis, and menstrual irregularities), but the questionnaire format between surveys differed, so comparisons could not be made. The frequency of occurrence of each symptom was generally similar between surveys. The frequency of fever and headache was slightly higher and that of sore throat was lower in the Atomic Bomb Disaster Survey than in the Joint Commission Survey. Agreement as assessed by the kappa coefficient [21] was relatively low for pigmentation and sore throat, but very high (> 0.80) and almost perfect [23] for the major radiation symptoms of vomiting, diarrhea, nausea, epilation, and nose bleeding.

**Table 3:** Comparison of symptom occurrence between the Atomic Bomb Disaster Survey and the US–Japan Joint Commission Survey (*n* = 3447)

No.	Symptom	A-Bomb Disaster Survey	(%)	Joint Commission Survey	(%)	Symptomatic matched^a^	(%)	No-symptomatic matched^b^	(%)	Kappa coefficient	95% confidence interval
1	Vomiting	660	(19.1)	649	(18.8)	617	(93.5)	2755	(98.9)	0.93	(0.91, 0.95)
2	Diarrhea	1286	(37.3)	1246	(36.1)	1199	(93.2)	2114	(97.8)	0.92	(0.90, 0.93)
3	Nausea	689	(20.0)	692	(20.1)	635	(92.2)	2701	(97.9)	0.90	(0.88, 0.92)
4	Epilation	513	(14.9)	455	(13.2)	432	(84.2)	2911	(99.2)	0.88	(0.85, 0.90)
5	Nasal bleeding	142	(4.1)	117	(3.4)	106	(74.6)	3294	(99.7)	0.81	(0.76, 0.86)
6	Fever	932	(27.0)	718	(20.8)	671	(72.0)	2468	(98.1)	0.76	(0.73, 0.78)
7	Cramps	55	(1.6)	52	(1.5)	38	(69.1)	3378	(99.6)	0.71	(0.61, 0.80)
8	Headache	820	(23.8)	529	(15.3)	511	(62.3)	2609	(99.3)	0.70	(0.67, 0.73)
9	Stomatitis/gingivitis	339	(9.8)	330	(9.6)	242	(71.4)	3020	(97.2)	0.69	(0.65, 0.74)
10	Petechiae	172	(5.0)	251	(7.3)	143	(83.1)	3167	(96.7)	0.66	(0.60, 0.71)
11	Purpura	125	(3.6)	135	(3.9)	83	(66.4)	3270	(98.4)	0.62	(0.55, 0.69)
12	Skin pigmentation	149	(4.3)	117	(3.4)	78	(52.3)	3259	(98.8)	0.57	(0.50, 0.64)
13	Sore throat	319	(9.3)	624	(18.1)	289	(90.6)	2793	(89.3)	0.56	(0.52, 0.60)

^a^Matched number of symptomatic cases between both surveys and proportion (%) for the A-Bomb Disaster Survey data.

^b^Matched number of no-symptomatic cases.

### Exclusion of data on radiation dose estimation

Table 4 presents the characteristics of the subjects for whom doses could be estimated and the percentage of exclusion by characteristic. The overall exclusion rate from all data was 38.3%, with the highest rate at 56.9% for distances <1 km from the hypocenter. A significant number of subjects who were exposed to radiation at close range were well shielded by bomb shelters, concrete, or brick buildings, making dose estimation impossible.

**Table 4:** Proportion of data exclusion by radiation dose estimation from the base population

Characteristic		Subjects with a dose available	(%)	Proportion of data excluded (%)	All survey subjects^a^	(%)
Status	Alive	3323	(93.2)	39.0	5446	(94.2)
	Deceased	243	(6.8)	27.0	333	(5.8)
Sex	Male	1510	(42.3)	42.2	2613	(45.2)
	Female	2056	(57.7)	34.8	3151	(54.5)
Age at the time of the	< 10	370	(10.4)	23.4	483	(8.4)
bombing (years)	10–19	1720	(48.2)	41.8	2953	(51.1)
	20–29	412	(11.6)	41.9	709	(12.3)
	30–39	300	(8.4)	37.0	476	(8.2)
	40–49	338	(9.5)	35.7	526	(9.1)
	50–59	251	(7.0)	36.1	393	(6.8)
	60–86	175	(4.9)	26.8	239	(4.1)
Distance from the	< 1.0	205	(5.7)	56.9	476	(8.2)
hypocenter (km)	1.0–1.4	632	(17.7)	50.4	1273	(22.0)
	1.5–1.9	685	(19.2)	39.2	1126	(19.5)
	2.0–2.9	1242	(34.8)	28.7	1741	(30.1)
	3.0–3.9	761	(21.3)	31.0	1103	(19.1)
	4.0	41	(1.1)	31.7	60	(1.0)
Shielding	Outdoors, open	430	(12.1)	0.0	430	(7.4)
	Outdoors, wooden house shielding	117	(3.3)	0.0	117	(2.0)
	Indoors, wooden house	3019	(84.7)	0.0	3019	(52.2)
	Unknown or others	0	(0.0)	100.0	2213	(38.3)
	Total	3566	(100.0)	38.3	5779	(100.0)

^a^Excluding 16 from among all 5795 survey subjects whose distance from the hypocenter was >4 km.

### Classification of trends in changes in the frequency of each symptom by dose

**Fig. 3:**
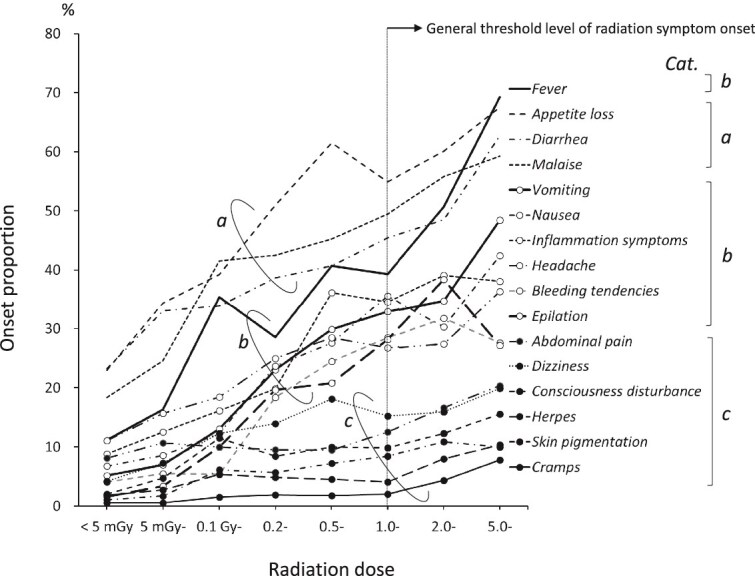
Categorization of symptoms by radiation dose response. Category a: Related to radiation dose also at lower doses than the threshold level and a relatively higher proportion even at the lowest dose. b: Related to radiation dose also at lower than the threshold level. c: Related to radiation dose only at above the threshold level.

Figure 3 shows the frequency of onset of 16 symptoms related to radiation dose in the Atomic Bomb Disaster Survey. Based on the trends in frequency of occurrence with radiation dose, these symptoms could be classified into three categories according to their frequency of occurrence around the lowest dose and their correlation (proportional relationship) with radiation dose. Category ***a*** has a relatively high incidence (~20%) at doses <5 mGy and is proportional to a dose even below the threshold (1 Gy). Category ***b*** has a low incidence at the lowest dose (≤ 10%) and is proportional to a dose even below the threshold. Category ***c*** is not proportional to a dose below the threshold, but is proportional to a dose above the threshold. The slopes for categories ***a*** and ***b*** are nearly equal. Category ***c*** symptoms, such as abdominal pain, dizziness, and consciousness disturbance, exhibited no correlation with subthreshold doses and an increased frequency for above-threshold doses. The frequency of inflammation symptoms, bleeding tendency, and epilation tended to be rather low in the highest dose category (≥ 5 Gy).

### Frequency of external injuries and burns by radiation dose

**Fig. 4:**
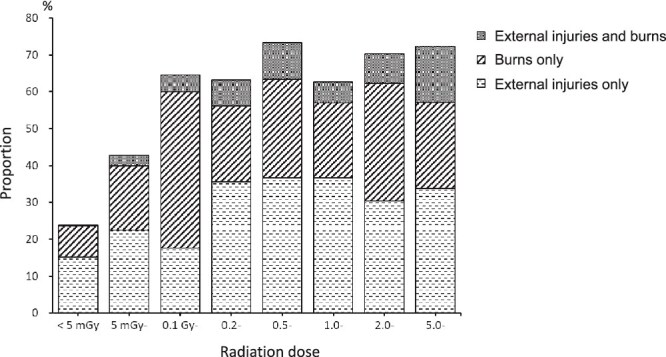
Proportion of external injuries and/or burns by radiation dose.

Of the 3566 subjects, 1049 (29.4%) had external injuries and 796 (22.3%) had burns. Figure 4 shows the frequency of external injuries and burns by radiation dose category. Blasts and heat rays, which cause external injuries and burns to the human body, should increase along with radiation dose with closer proximity to the hypocenter, but the total frequency (~70%) of the three groups—external injuries only, burns only, and both—did not change at doses >0.1 Gy. The frequency of burns only was highest at doses of 0.1–0.2 Gy.

### Odds ratios of symptom onset due to radiation, external injuries, and burns

**Fig. 5:**
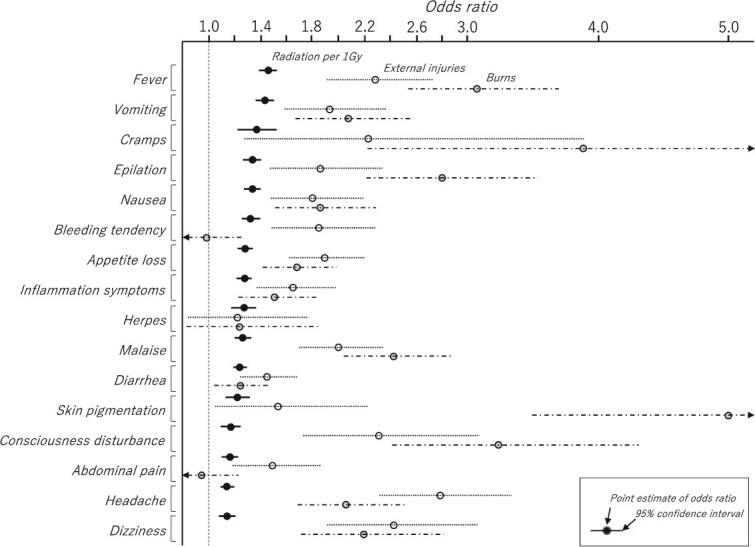
Odds ratios and 95% confidence intervals for radiation dose, external injuries, and burns for each symptom onset.

The analysis of the ORs for radiation dose per 1 Gy, external injuries, and burns adjusted for sex and age ATB for the occurrence of each symptom is shown in Fig. 5. Burns were strongly associated with fever, cramps, epilation, skin pigmentation, and consciousness disturbance, and the ORs for these symptoms were 3.06 (95% confidence interval: 2.54–3.70), 3.88 (2.22–6.77), 2.80 (2.21–3.54), 5.00 (3.49–7.16), and 3.23 (2.41–4.31), respectively. By contrast, bleeding tendency, herpes, and abdominal pain were not significantly associated with burns. Herpes was not associated with external injuries and burns and only showed a statistically significant association with radiation. The OR for radiation per 1 Gy was 1.27 (1.17–1.38) With the exception of cramps, most symptoms were statistically significantly associated with radiation dose after adjustment for sex, age ATB, external injuries, and burns, with particularly strong associations for fever and vomiting with ORs for radiation per 1 Gy of 1.46 (1.39–1.53) and 1.44 (1.37–1.51), respectively. The sex- and age ATB-adjusted ORs for radiation per 1 Gy without consideration of external injuries and burns were all slightly higher than the ORs with consideration of external injuries and burns, e.g. 1.53 (1.46–1.61) vs. 1.46 (1.39–1.53) for fever, 1.49 (1.43–1.57) vs. 1.43 (1.36–1.50) for vomiting, 1.47 (1.33–1.62) vs. 1.37 (1.23–1.52) for cramps, and 1.40 (1.34–1.47) vs. 1.34 (1.27–1.41) for epilation, respectively. In the analysis that included sex, age ATB, radiation, external injuries, and burns as factors, the ORs for the occurrence of symptoms by sex (male: 1 vs. female: 0) were as follows: 0.62 (0.51–0.75) for vomiting, 0.71 (0.59–0.86) for nausea, 0.80 (0.65–0.99) for bleeding tendency, 0.82 (0.71–0.95) for appetite loss, and 0.73 (0.61–0.88) for inflammation symptoms, which were more common in females, compared with 1.60 (1.13–2.27) for herpes, which was more common in males. When sex was included and excluded from the analysis model, the ORs for radiation symptoms were 1.46 (1.39–1.53) vs. 1.46 (1.39–1.53) for fever, 1.44 (1.37–1.51) vs. 1.43 (1.36–1.50) for vomiting, 1.37 (1.23–1.52) vs. 1.37 (1.23–1.52) for cramps, and 1.34 (1.27–1.41) vs. 1.34 (1.27–1.41) for epilation, respectively, with little difference between the sexed and unsexed models, including other symptoms.

### Possible selection for radiation-induced epilation

**Fig. 6:**
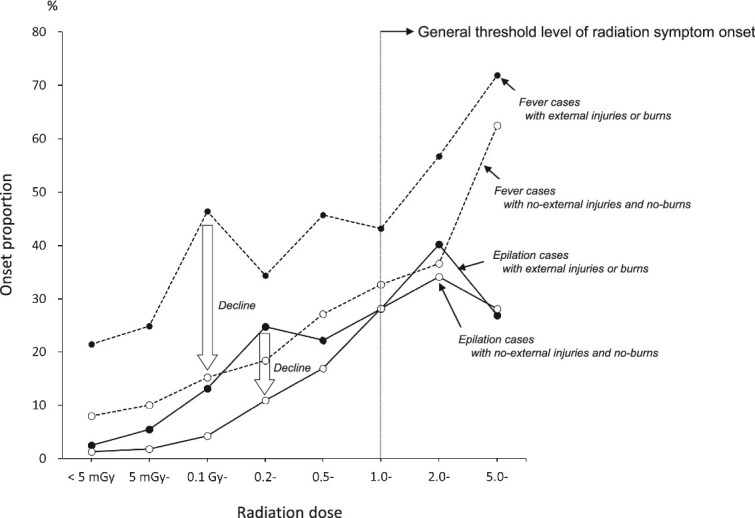
Proportion of fever and epilation onsets with and without external injuries and burns.

To examine more simply the effect of external injuries and burns on symptom expression, Fig. 6 shows the frequency of fever and epilation with and without external injuries and burns at each exposure dose. The group without external injuries or burns (*n* = 1877) had a lower frequency of both fever and epilation than the group with external injuries or burns (*n* = 1689). In the group with external injuries or burns, the frequency of fever at doses <5 mGy was >20%, while in the group without external injuries or burns, the frequency was <10%, with remarkably low frequencies across all dose categories. However, the frequency of symptoms without external injuries or burns was proportional to the radiation dose, even at subthreshold levels.

We also investigated the possibility of selecting radiation-induced epilation by time of onset, duration, and severity. Radiation-induced epilation is known to be caused by temporary damage to hair matrix cells, which undergo active cell division. In most cases, hair loss occurs 3–4 weeks after radiation exposure, followed by recovery [24]. Regarding the severity of epilation, Table 5 classifies all epilation cases and severe epilation (‘all or many’ as a response to the questionnaire) by time of onset and duration, classified into doses of <1 Gy and ≥ 1 Gy. Onset was most common within 3 weeks (by August 31) for both dose categories. In the case of severe epilation, the most common time of onset was 2–3 weeks later (21–31 August). The duration of epilation was usually within 1 month of onset. In the case of severe epilation and exposure to ≥1 Gy, epilation did not occur for > ~5 weeks.

**Table 5:** Classification of the number of epilations by radiation exposure dose, date of onset and duration

a. All cases of epilation								
	Dose <1 Gy				Dose ≥1 Gy			
	Duration				Duration			
Onset date	< 1 week	1 week to 1 month	1 month or over	Total	< 1 week	1 week to 1 month	1 month or over	Total
9–20 Aug. (1–2 weeks)	8 (6.3)	18 (14.2)	13 (10.2)	39 (30.7)	13 (9.8)	20 (15.0)	14 (10.5)	47 (35.3)
21–31 Aug. (2–3 weeks)	10 (7.9)	11 (8.7)	9 (7.1)	30 (23.6)	17 (12.8)	21 (15.8)	9 (6.8)	47 (35.3)
1–10 Sep. (3–4 weeks)	5 (3.9)	9 (7.1)	8 (6.3)	22 (17.3)	6 (4.5)	13 (9.8)	4 (3.0)	23 (17.3)
11–20 Sep. (4–5 weeks)	3 (2.4)	2 (1.6)	3 (2.4)	8 (6.3)	2 (1.5)	2 (1.5)	3 (2.3)	7 (5.3)
21–30 Sep.	1 (0.8)	3 (2.4)	1 (0.8)	5 (3.9)	0 (0.0)	0 (0.0)	1 (0.8)	1 (0.8)
1 Oct. or later	3 (2.4)	14 (11.0)	6 (4.7)	23 (18.1)	3 (2.3)	4 (3.0)	1 (0.8)	8 (6.0)
Total	30 (23.6)	57 (44.9)	40 (31.5)	127 (100.0)	41 (30.8)	60 (45.1)	32 (24.1)	133 (100.0)
b. Severe epilation (total or substantial hair loss)								
	Dose <1 Gy				Dose ≥1 Gy			
	Duration				Duration			
Onset date	< 1 week	1 week to1 month	1 month or over	Total	< 1 week	1 week to 1 month	1 month or over	Total
9–20 Aug. (1–2 weeks)	0 (0.0)	6 (20.7)	4 (13.8)	10 (34.5)	5 (7.9)	13 (20.6)	7 (11.1)	25 (39.7)
21–31 Aug. (2–3 weeks)	5 (17.2)	6 (20.7)	1 (3.4)	12 (41.4)	10 (15.9)	11 (17.5)	7 (11.1)	28 (44.4)
1–10 Sep. (3–4 weeks)	0 (0.0)	0 (0.0)	1 (3.4)	1 (3.4)	0 (0.0)	7 (11.1)	1 (1.6)	8 (12.7)
11–20 Sep. (4–5 weeks)	0 (0.0)	1 (3.4)	1 (3.4)	2 (6.9)	0 (0.0)	1 (1.6)	1 (1.6)	2 (3.2)
21–30 Sep.	0 (0.0)	0 (0.0)	0 (0.0)	0 (0.0)	0 (0.0)	0 (0.0)	0 (0.0)	0 (0.0)
1 Oct. or later	0 (0.0)	3 (10.3)	1 (3.4)	4 (13.8)	0 (0.0)	0 (0.0)	0 (0.0)	0 (0.0)
Total	5 (17.2)	16 (55.2)	8 (27.6)	29 (100.0)	15 (23.8)	32 (50.8)	16 (25.4)	63 (100.0)

### Combination of symptoms

**Fig. 7:**
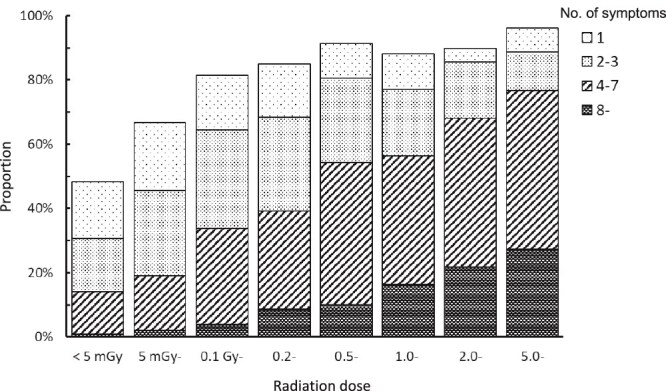
Proportion of subjects by number of simultaneous symptom onset and radiation dose.

Figure 7 shows the percentage of subjects with symptoms for each radiation dose category, classified by the number of symptoms that occurred simultaneously. In total, ≥ 80% of the subjects in the ≥0.1 Gy dose category had some symptoms. The percentage of subjects who presented with four or more symptoms increased with radiation dose. Four or more symptoms are observed in >50% of the subjects at doses of ≥1 Gy. A higher number of symptoms indicates a higher dose.

**Fig. 8:**
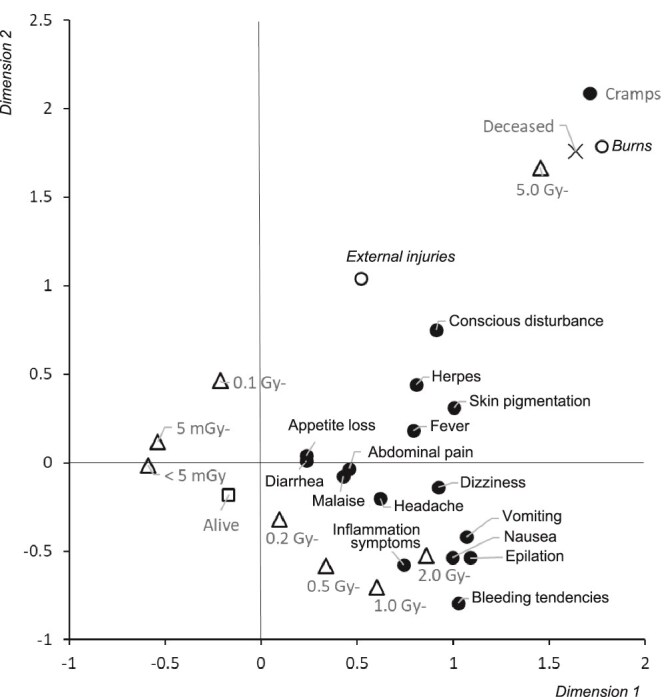
Spatial relationships among 16 symptoms, external injuries and burns, life-and-death status, and radiation dose.

Figure 8 shows the results of a multiple correspondence analysis of the association between radiation dose, life and death, external injuries and burns, and 16 symptoms. The closer the distance between each factor, the stronger the association. The horizontal axis, dimension 1, can be thought of as the radiation dose in approximate orders of magnitude, from left to right. To the left of the origin are survivors and < 5 mGy, 5–100 mGy, and 0.1–0.2 Gy. No association was observed between these doses and deaths. In terms of symptoms, the distance between appetite loss, diarrhea, abdominal pain, and malaise was very close, and was also close to doses <1 Gy. In addition, they were relatively far from external injuries and burns. Nausea, vomiting, and epilation were close together, and inflammation symptoms and bleeding tendency were also relatively close together around doses between ≥2 and < 5 Gy. Cramps, deaths, burns, and exposure to doses >5 Gy were close together.

## DISCUSSION

In this study, we conducted a multidimensional analysis of radiation-induced symptom expression based on data obtained from a survey conducted immediately after the atomic bombing in Nagasaki, which minimized recall bias, and evaluated the possibility of identifying radiation-induced symptoms among these symptoms.

### Comparison of the results of the two surveys

Both the Atomic Bomb Disaster Survey and the US–Japan Joint Commission Survey were conducted by medical personnel at a very early date, 2 or 3 months after the bombing. Many of the symptoms could not be objectively confirmed, as they were considered to have disappeared by the time of the survey, but were based primarily on patients’ reports, including their memories. The patients themselves may not have known whether the symptoms they experienced were caused by radiation or other causes. In our previous study, in a comparison of surveys on acute symptoms conducted immediately after the bombing and several decades later, the typical radiation symptoms of epilation and subcutaneous bleeding tended to increase in the later survey, and this was thought to be due to memory modification [24]. In the present study, a comparison of the results of the surveys immediately after the bombing showed that the responses reported for the symptoms of nausea, vomiting, diarrhea, nosebleeds, and epilation were almost perfectly consistent (Table 3). These symptoms are particularly common with high-dose radiation exposure. Fever tends to have slightly lower agreement, which may be related to the fact that it is a common symptom also seen in a variety of infectious diseases. Therefore, these surveys were considered to have had sufficient reliability.

### Classification of trends in the changes in the frequency of each symptom by dose

Regarding the frequency of symptoms by dose category (Fig. 3), the frequency of inflammation symptoms, bleeding tendency, and epilation tended to be lower in the highest dose category (≥ 5 Gy). The frequencies of inflammation symptoms exposed to ≥5 Gy (34.7% and 40.6% in survivors and deceased subjects, respectively) and bleeding tendency (29.6% and 42.9%, respectively) were higher in deceased subjects, whereas the frequency of epilation was higher in survivors (36.7% and 20.3% in survivors and deceased subjects, respectively). The onset of epilation increased after 2–3 weeks and may not have been observed in earlier deaths.

Inflammation symptoms and bleeding tendency were more common among the deceased for radiation exposure doses ≥5 Gy, suggesting that those who experienced these symptoms died early and were not included in the survey. It is well known that radiation-induced acute symptoms occur as a tissue reaction at a threshold dose of ~1 Gy [16, 17]. Because of the existence of threshold doses, these symptoms may include many symptoms caused by factors other than radiation, such as external injuries and burns. In particular, category ***a*** symptoms (appetite loss, diarrhea, and malaise, etc.) were present at a frequency of ~20% even at doses <5 mGy, suggesting that they may be due to factors other than radiation. The overall frequency was high. The high overall frequency of these symptoms may be due to psychological effects, such as feelings of helplessness, abandonment due to the end of the war, and the loss of relatives and family members. Category ***b*** symptoms (e.g. fever, vomiting, nausea, inflammation symptoms) may be caused by factors other than radiation, such as infectious diseases resulting from poor living conditions. A proportional relationship between dose and frequency was also observed in the subthreshold dose range in these symptom categories, which may have been influenced by the fact that the closer one was to the hypocenter, the more often one lost close relatives and homes one had lived with, and this may have increased the sense of loss and deterioration of the living environment.

### Association of symptom onset with radiation, external injury, and burns, and case selection for radiation symptoms

The reliability of the data for external injuries and burns is considered to be relatively high because many of these injuries are likely to have been objectively observed by the investigator at the time of the survey. The frequency of external injuries and burns within ~2 km of the hypocenter (> 0.1 Gy) was high, at around 70%, and did not change for higher dose categories (Fig. 4). The frequency of burns only was highest at 0.1–0.2 Gy. This dose is equivalent to exposure inside a Japanese house ~1.7–1.8 km from the hypocenter and may include burns from the fire of a collapsed house. People with these injuries are at increased risk for infectious diseases that can cause fever and other symptoms. The ORs for symptoms due to burns were particularly high for fever, cramps, epilation, skin pigmentation, and consciousness disturbance (Fig. 5). Fever due to burn infection and epilation and skin pigmentation due to head burns were difficult for patients themselves to distinguish from radiation symptoms. External injuries, as well as burns, were strongly associated with the development of each symptom. Fever due to infection resulting from external injuries and epilation due to head injury were considered possible causes. The ORs for radiation per 1 Gy adjusted for external injuries and burns were lower than the ORs without adjustment for each symptom. External injuries and burns were confounding factors for the development of radiation-related symptoms. A marked decrease was seen in the frequency of fever and hair loss in the absence of external injuries and burns, but the tendency to increase with radiation dose remained for doses even <1 Gy (Fig. 6), which strongly suggests that there are causes other than external injuries and burns.

It is possible to select the extent of radiation-induced epilation by classifying epilation according to severity, time of onset, and duration of symptoms, but it seems difficult to distinguish clearly between radiation- and non-radiation-induced symptoms (Table 5). In addition, it is not appropriate to exclude subjects with external injuries and burns from the analysis, especially because these subjects may have been exposed to substantial amounts of radiation and may have developed symptoms. If possible, it would be important for the symptoms to be diagnosed or medically determined to be the result of radiation exposure at the time of the survey.

Because sex was not included in the survey data for the present study, it was estimated from names and other information and used in the analysis. The results of the OR analysis were similar in the models with and without sex, which suggests that sex had no effect on symptom expression.

### Distribution of the number of simultaneous symptom onsets by radiation dose

Even at doses of <0.1 Gy, 10%–20% of four or more symptoms have been observed (Fig. 7), because 0.1 Gy is equivalent to unshielded exposure at ~2 km from the hypocenter or inside a wooden house at 1.8–1.9 km, at which distance, wooden structures would be completely destroyed and heat energy sufficient to start a fire would reach the area. Radiation-induced symptoms should not be present when the estimated radiation dose is <0.1 Gy.

### Combination of radiation-induced symptoms

According to the treatment records of atomic bomb survivors [25], nausea, vomiting, and appetite loss as radiation-specific symptoms were seen from the day of the bombing, epilation began at ~14–21 days after the bombing, fever occurred a little later, ~19–28 days after the bombing, followed by inflammation symptoms such as bleeding spots on the skin and gingivitis due to bone marrow damage and subsequently bleeding tendency such as bleeding from the gums and nasal bleeding due to bone marrow disorder. It is thought that a number of such symptoms can be seen when exposed to a certain amount of radiation. On the other hand, as an example of damage caused by radiation alone, which is different from that resulting from the atomic bomb, the record of a victim of a radiation exposure accident at a nuclear fuel processing plant in Japan in 1999 [26, 27] shows that nausea and vomiting began a few hours after exposure, followed by lymphocytopenia, fatigue, and skin loss (Day 11), diarrhea, and bleeding (Day 27). The symptoms of radiation exposure were observed as a series. On the other hand, the Atomic Bomb Casualty Survey, conducted several months after the bombing, did not provide sufficient answers about the timing of the onset of symptoms, and those experienced at the time of the survey were a combination of symptoms. The symptoms recorded in the survey included those caused by a variety of factors, including non-radiation external injuries and burns.

The presence of a combination of symptoms seen with high-dose radiation exposure, such as epilation, nausea, vomiting, and bleeding, and the large number of these symptoms that occurred, likely indicates a high radiation dose (Fig. 8). Thus, when symptoms are the result of radiation exposure, they are often observed in combination with other symptoms, and a higher number of symptoms may indicate a higher radiation dose. This may help to distinguish whether symptoms are due to radiation exposure.

As noted above, it may not be appropriate to use symptom data alone as a factor related to radiation exposure for the analysis of radiation effects because symptom data obtained from interviews such as those used in the present study include symptoms due to factors other than radiation. This could be improved by using the presence or absence of symptom combinations seen at high doses of radiation exposure as an indicator of the certainty of radiation-induced symptom occurrence.

### Limitations

This study does have some limitations. First, information regarding deceased individuals was obtained from surviving family members or relatives. It is possible that the validity of this information is lower than that obtained from the person himself/herself.

Second, the subjects situated in relatively close proximity to the hypocenter were excluded from the analysis (Table 4). It was not possible to estimate the radiation dose, as these subjects would have been situated within well-shielded buildings, such as concrete or brick structures or bomb shelters. The exclusion of these subjects may have resulted in an underestimation of the frequency of symptoms observed at relatively higher doses or an underestimation of ORs for the occurrence of symptoms associated with the radiation dose. We have attempted to estimate ORs excluding the effects of external injuries and burns by conducting an analysis that incorporates these factors. Nevertheless, there still appears to be some confounding factors, such as hygienic, nutritional, and psychological factors, that cannot be excluded.

Third, the method used to estimate radiation doses is simplistic and may be subject to misclassification, especially in the absence of detailed shielding information. The OR for the onset of radiation symptoms itself requires careful evaluation.
